# Ligands with Two Monoanionic N,N‐Binding Sites: Synthesis and Coordination Chemistry

**DOI:** 10.1002/chem.201903442

**Published:** 2019-11-22

**Authors:** Robert Kretschmer

**Affiliations:** ^1^ Junior Professorship Inorganic Chemistry of Catalysis, Institute of Inorganic and Analytical Chemistry Friedrich Schiller University Jena Humboldtstrasse 8 07743 Jena Germany; ^2^ Jena Center for Soft Matter (JCSM) Friedrich Schiller University Jena Philosophenweg 7 07743 Jena Germany

**Keywords:** amidines, dinuclear complexes, dinucleating ligands, guanidines, mononuclear complexes

## Abstract

Polytopic ligands have become ubiquitous in coordination chemistry because they grant access to a variety of mono‐ and polynuclear complexes of transition metals as well as rare‐earth and main‐group elements. Nitrogen‐based ditopic ligands, in which two monoanionic N,N‐binding sites are framed within one molecule, are of particular importance and are therefore the primary focus of this review. In detail, bis(amidine)s, bis(guanidine)s, bis(β‐diimine)s, bis(aminotroponimine)s, bis(pyrrolimine)s, and miscellaneous bis(N,N‐chelating) ligands are reviewed. In addition to the general synthetic protocols, the application of these ligands is discussed along with their coordination chemistry, the multifarious binding modes, and the ability of these ligands to bridge two (or more) metal(loids).

## Graphical abstract

**N,N Ligands in the spotlight**: Whether mono‐ or polynuclear complexes are derived from nitrogen‐based ditopic ligands with two monoanionic N,N‐binding sites depends on several parameters related to the ligand and the metal(loid) itself.

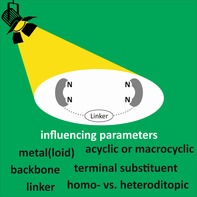

## 1 Introduction

**Figure 1:**
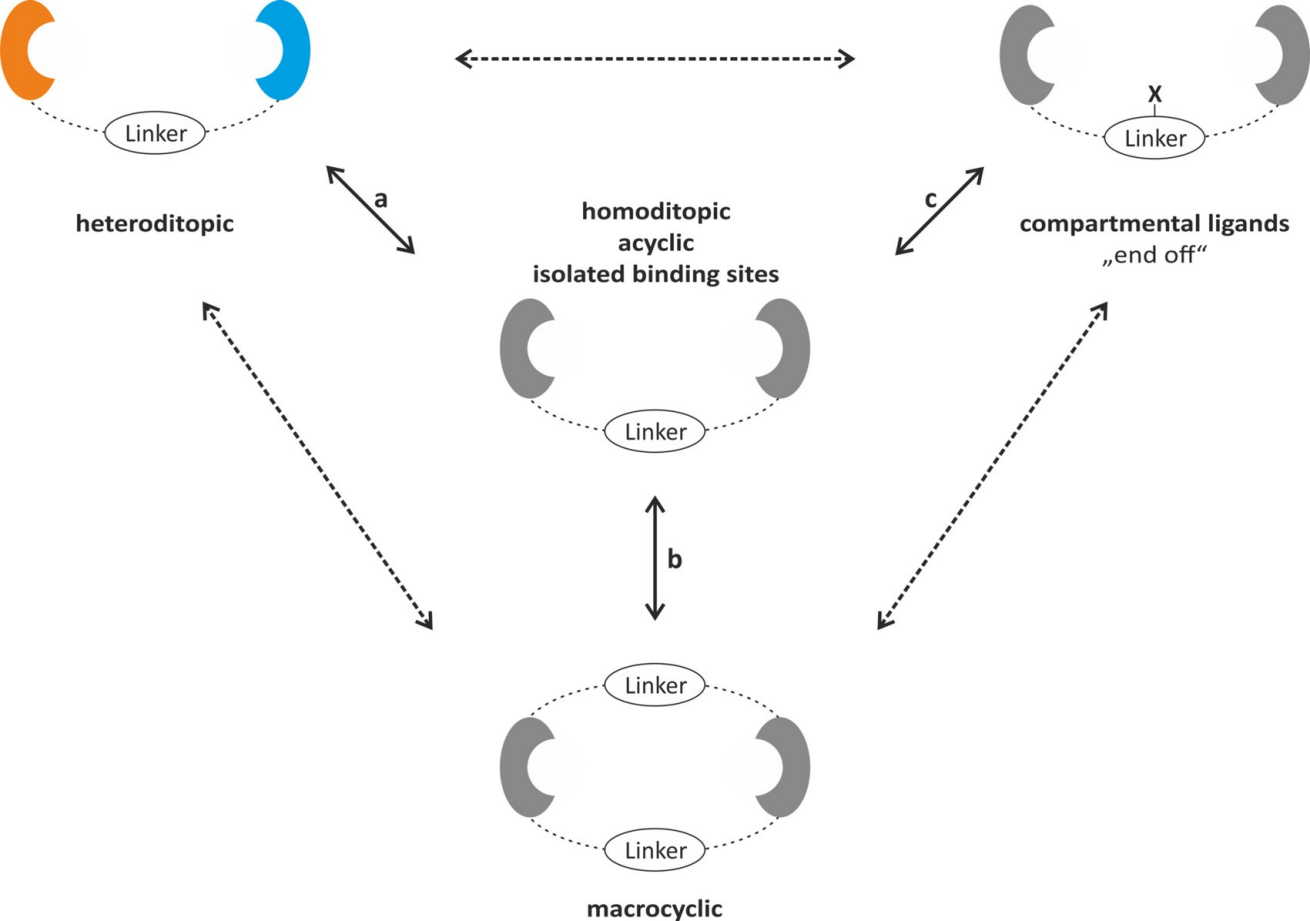
Schematic representation of dinucleating ligands: a) hetero‐ versus homoditopic, b) acyclic versus macrocyclic, and c) compartmental ligands (X=bridging donor atom) and ligands with isolated binding sites.

Various fields of modern coordination chemistry are affected by polydentate ligands, which give rise to an increased stability of the related complexes and allow for a fine tuning of the reactivity and selectivity of the active center(s) through their steric and electronic properties. In addition, polydentate ligands enable the synthesis of various types of compounds, ranging from stable, sterically shielded mononuclear metal complexes to intricate polynuclear species.^1^ Recently, the latter complexes received particular attention due to the increasing interest in cooperative effects^2^ associated with polynuclear metal(loid) complexes and, in this regard, anionic nitrogen‐based ligands have become valuable supports. With respect to cooperative effects, ditopic ligands play a crucial role not only for adjusting the metal(loid)–metal(loid) separation, which amounts preferentially to 3.5–6 Å,^3^ but also for directing the two metal(loid) centers in a cooperative manner.^2^ In addition to their use as frameworks for homo‐ and heterodinuclear complexes, ditopic ligands are also used as molecular sensors, (super)bases, or organocatalysts.^4^ Since the first reports on dinucleating ligands,^5^ the concept has gained substantial scientific interest with numerous different types known today and several classifications are used in the literature. At first, they can be divided into homoditopic and heteroditopic ligands, depending on whether the ligand frames two binding sites of the same or of different type, respectively (Figure 1 a). The embedding of the binding sites in either an acyclic or macrocyclic framework allows for another differentiation (Figure 1 b). Finally, an additional classification in compartmental ligands and ligands with isolated binding site is made depending on the availability of donor atoms bridging both binding sites (Figure 1 c). As will be shown in this review, the differentiation of a ligand as either a ditopic or a tetradentate ligand without considering the central atom is often not possible as the formation of a mono‐, di‐, or polynuclear complex strongly depends on the interplay of both the metal(loid) and the ligand. This review covers the synthesis and application of dinucleating ligands with two monoanionic N,N‐binding sites, whereas it is limited to ligands in which the metal(loid) ligand binding is achieved primarily through the nitrogen atoms. The chapters belong to bis(amidine)s, bis(guanidine)s, bis(β‐diimine)s, bis(aminotroponimine)s, bis(pyrrolimine)s, and miscellaneous ligands. Every chapter starts with the synthetic access of the related ligands and is followed by a section discussing their application and coordination chemistry.

## 2 Bis(amidine)s

Bis(amidine)s, which have been synthesized in 1937 for the first time,^6^ can be divided into two classes according to the way the two amidine units are connected, these being through the nitrogen atom or through the backbone‐based carbon atom, and both types will be discussed sequentially in the following. Persubstituted bis(amidine)s, which in consequence do not bear acidic protons, are beyond the scope of this review and have been excluded.

**Scheme 1:**
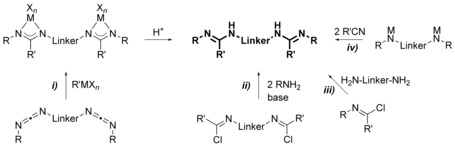
Synthesis of N‐bridged bis(amidine)s.

N‐Bridged bis(amidine)s are in general accessible on the four routes depicted in Scheme 1: *i*) treatment of a bis(carbodiimide) with organometallic compounds,^7^ synthesis via *ii*) bis(imidoylchloride)s^6^, ^8^ and *iii*) imidoylchlorides,7d, 9 respectively, or *iv*) by the addition of nitriles to metallated secondary diamines;^10^ Table 1 summarizes all N‐bridged bis(amidine)s reported so far.

**Table 1:** Known N‐bridged bis(amidine)s of the depicted type.

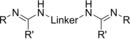									
Linker	R	R′	Method^[a]^	Ref.	Linker	R	R′	Method^[a]^	Ref.
‐C_2_H_4_‐	C_6_H_5_	CH_3_	*ii*	8a	*trans*‐1,2‐C_6_H_10_‐	C_6_H_5_	C(CH_3_)_3_	*ii*	8d
‐C_2_H_4_‐	C_6_H_5_	C_6_H_5_	*ii*	8a	*trans*‐1,2‐C_6_H_10_‐	2‐C_5_H_4_N	C(CH_3_)_3_	*ii*	8d
‐C_2_H_4_‐	C_6_H_5_	(C_2_H_7_)_2_CH	*ii*	8a	*trans*‐1,2‐C_6_H_10_‐	3,5‐(CH_3_)_2_C_6_H_3_	C(CH_3_)_3_	*ii*	8d
‐C_2_H_4_‐	*p‐*C_2_H_5_OC_6_H_4_	CH_3_	*ii*	8a	*trans*‐1,2‐C_6_H_10_‐	2,6‐(CH{CH_3_}_2_)_2_C_6_H_3_	C(CH_3_)_3_	*ii*	8d
‐C_2_H_4_‐	*p‐*C_2_H_5_OC_6_H_4_	(C_2_H_5_)_2_CH	*ii*	8a	*trans*‐1,2‐C_6_H_10_‐	2,5,6‐(CH_3_)_3_C_6_H_2_	C(CH_3_)_3_	*ii*	8d
‐C_2_H_4_‐	*p‐*C_2_H_5_OC_6_H_4_	(C_2_H_7_)_2_CH	*ii*	8a	*trans*‐1,2‐C_6_H_10_‐	C_6_H_5_	C_6_H_5_	*ii*	8b
‐C_2_H_4_‐	*p‐*C_2_H_5_OC_6_H_4_	CH_3_(CH_2_)_5_CH_2_	*ii*	8a	*trans* −1,2‐C_6_H_10_‐	C_6_H_5_	*p‐*C(CH_3_)_3_C_6_H_4_	*ii*	8b
‐C_2_H_4_‐	*p‐*C_2_H_5_OC_6_H_4_	C_6_H_5_	*ii*	8a	*trans* −1,2‐C_6_H_10_‐	C_6_H_5_	*p‐*CH_3_C_6_H_4_	*ii*	8b
‐C_2_H_4_‐	*p‐*C_2_H_5_OC_6_H_4_	C_6_H_5_CH_2_CH_2_	*ii*	8a	*trans* −1,2‐C_6_H_10_‐	*p‐*CH_3_OC_6_H_4_	*p‐*C(CH_3_)_3_C_6_H_4_	*ii*	8b
‐C_2_H_4_‐	*p‐*C_2_H_5_OC_6_H_4_	C_6_H_5_OCH_2_	*ii*	8a	*trans* −1,2‐C_6_H_10_‐	*p‐*CH_3_C_6_H_4_	*p‐*CH_3_C_6_H_4_	*ii*	8b
‐C_2_H_4_‐	*p‐*C_2_H_5_OC_6_H_4_	CH_3_/C_6_H_5_^[b]^	*ii*	8a					
‐C_2_H_4_‐	*p‐*C_2_H_5_CO_2_C_6_H_4_	CH_3_	*ii*	8a	1,8‐naphtyl	2,6‐(CH_3_)_2_C_6_H_3_	C(CH_3_)_3_	*ii*	8f
‐C_2_H_4_‐	2,4,6‐(CH_3_)_3_C_6_H_2_	C_6_H_5_	*ii*	8h					
					‐Si(CH_3_)_2_‐	C(CH_3_)_3_	C_6_H_5_	*v*	13
‐C_3_H_6_‐	*p‐*CH_3_C_6_H_4_	C_6_H_5_	*ii*	8e					
‐C_3_H_6_‐	*p‐*C(CH_3_)_3_C_6_H_4_	C_6_H_5_	*ii*	8e	4,4′‐benzidine	2,6‐(CH{CH_3_}_2_)_2_C_6_H_3_	CH_3_	*iii*	9
‐C_3_H_6_‐	*p‐*CH_3_OC_6_H_4_	C_6_H_5_	*ii*	8e	4,4′‐azobenzene	2,6‐(CH{CH_3_}_2_)_2_C_6_H_3_	CH_3_	*iii*	9
									
‐C_5_H_10_‐	2,6‐(CH{CH_3_}_2_)_2_C_6_H_3_	CH_3_	*i*	7d	‐C_3_H_6_‐	Si(CH_3_)_3_	C_6_H_5_	*iv* ^[c]^	10a
‐C_5_H_10_‐	2,6‐(CH{CH_3_}_2_)_2_C_6_H_3_	C(CH_3_)_3_	*iii*	7d	‐1,2‐C_6_H_4_‐	Si(CH_3_)_3_	C_6_H_5_	*iv* ^[c]^	10e
‐C_5_H_10_‐	2,6‐(CH{CH_3_}_2_)_2_C_6_H_3_	CF_3_	*iii*	7d	‐1,3‐C_6_H_4_‐	Si(CH_3_)_3_	C_6_H_5_	iv^[c]^	10e
‐C_5_H_10_‐	2,6‐(CH{CH_3_}_2_)_2_C_6_H_3_	C_6_H_5_	*iii*	7d	*trans*‐1,2‐C_6_H_10_‐	Si(CH_3_)_3_	C_6_H_5_	*iv* ^[c]^	10b, 10c
‐C_5_H_10_‐	2,6‐(CH_3_)_2_C_6_H_3_	CH_3_	*i*	7d	‐Si(CH_3_)_2_‐	C(CH_3_)_3_	C_6_H_5_	*iv* ^[c]^	10d
					‐Si(CH_3_)_2_‐	C_6_H_5_	C_6_H_5_	*iv* ^[c]^	10d
‐1,2‐C_6_H_4_‐	2,6‐(CH_3_)_2_C_6_H_3_	C(CH_3_)_3_	*ii*	8g	‐Si(CH_3_)_2_‐	2,6‐(CH_3_)_2_C_6_H_3_	C_6_H_5_	*iv* ^[c]^	10d
‐1,2‐C_6_H_4_‐	2,6‐(CH{CH_3_}_2_)_2_C_6_H_3_	C(CH_3_)_3_	*ii*	8d, 8g	‐Si(CH_3_)_2_‐	2,6‐(CH_3_)_2_C_6_H_3_	C_6_H_5_	*iv* ^[d]^	10d
‐1,2‐C_6_H_4_‐	2,4,6‐(CH_3_)_3_C_6_H_2_	C(CH_3_)_3_	*ii*	8d	‐Si(CH_3_)_2_‐	2,6‐(CH{CH_3_}_2_)_2_C_6_H_3_	C_6_H_5_	*iv* ^[c]^	10d

[a] See Schemes 1, 3, and 4. [b] Heteroditopic ligand. [c] Only isolated as lithium salt. [d] Only isolated as sodium salt.

**Scheme 2:**
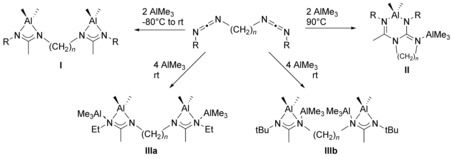
Reaction of bis(carbodiimide)s with trimethylaluminum under various conditions. *n*=3, 4; R=*t*Bu, Et, Ph.7c

Due to the limited stability of bis(carbodiimide)s,7c, 11 which are prone to polymerization reactions, the first route is restricted to only a few examples. However, it offers the advantage that the respective dinuclear aluminum,7c titanium,7a and zirconium7b complexes are directly obtained in yields >80 % without the isolation of the respective bis(amidine). Notably, for the reactions of bis(carbodiimide)s with trimethylaluminum, different products are obtained, depending on both the stoichiometry and the reaction conditions (Scheme 2).7c Although using a 1:2 ratio of bis(carbodiimide) and trimethylaluminum at low temperatures (−80 °C→rt) gives rise to dinuclear complexes **I**, increasing the temperature yields the dinuclear heterocycle **II**, and using a 1:4 ratio at ambient temperature results in the tetranuclear bis(amidinate) complexes **IIIa** or **IIIb**, depending on the steric bulk of the terminal substituent.

The use of imidoylchlorides and bis(imidoylchloride)s is the most general approach towards the synthesis of N‐bridged bis(amidine)s and this method has also been used to synthesize the first reported example back in the 1930s.^6^ Most of the synthetic protocols make use of bis(imidoylchloride)s and primary amines (route *ii*),^6^, ^8^ whereas there is only limited precedence for routes starting from imidoylchlorides and primary diamines (route *iii*, Scheme 1).7d, 9 In the first case, reactions are regularly carried out in toluene using two equivalents of the respective aniline and one equivalent of the bis(imidoylchloride) providing yields of isolated materials ranging from only a few percent to nearly quantitative product formation. It is worth noting that early reports by Hill and Johnston indicate that an excess of aniline is not only unnecessary, but also undesirable because the excess complicates the purification of the bis(amidine)s.8a Although alkyl‐bridged bis(imidoylchloride)s do not require the addition of a base, in case of bis(imidoylchloride)s containing aromatic bridges, triethylamine has been used in all synthesis reported so far. For the second route which uses imidoylchlorides and primary diamines, yields ranging from about 30 to 80 % have been reported, and performing the reaction in toluene at elevated temperatures seems to be preferential. Although further general conclusions remain elusive due to the limited sample set, it appears that the product yield is more affected by the electronic and steric properties of both the imidoylchloride and the primary diamine, rather than the experimental protocol, that is performing the reaction with or without triethylamine as an HCl scavenger. Notably, a 2,6‐pyridylene bridged bis(amidine) was obtained in 66 % yield starting from dilithiated 2,6‐diaminopyridine and the respective imidoylchloride.^12^ In conclusion, both routes appear to be suitable and the preference for one over the other is most likely due to the synthetic aim: for ligand libraries with varying terminal (R) or backbone (R′) substituents, the use of bis(imidoylchloride)s through route *ii*) is more convenient, whereas aiming for a ligand set with various linker groups, route *iii*) is of benefit.

**Figure 2:**
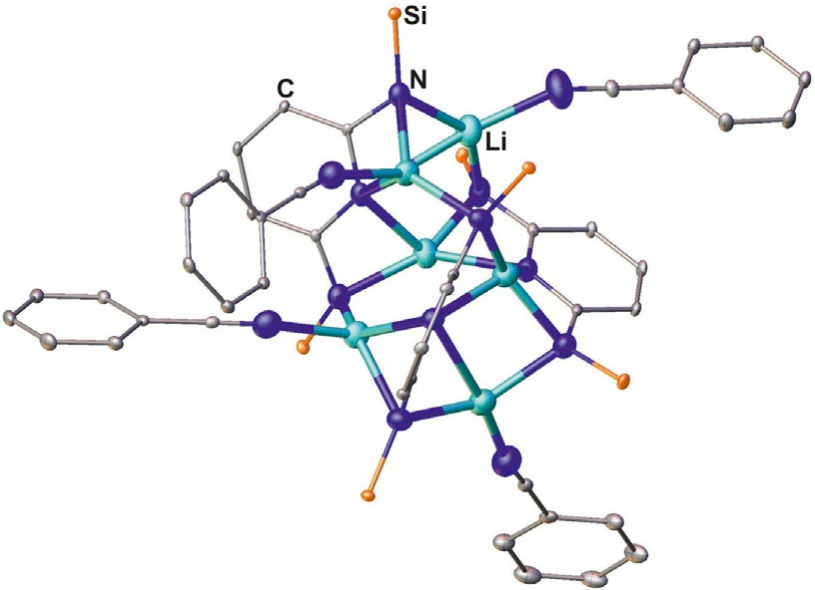
Molecular structure of **IV** in the solid state.10e Hydrogen atoms, solvent molecules, and methyl rests of the Si(CH_3_)_3_ groups are omitted for clarity.

A less common route is the reaction of lithiated secondary diamines with two equivalents of benzonitrile,^10^ which allows for the isolation of the related lithium bis(amidinate) complexes in yields ranging from 54 to 93 %. Even though these are suitable precursors for the synthesis of other metal complexes, this procedure has not been used so far to access the related bis(amidine)s. Although aliphatic, aromatic, and dimethylsilyl bridges have been applied, using a 2,6‐pyridylene bridging group does not afford the expected dinuclear bis(amidinate) complex, but yields the hexanuclear lithium complex **IV** bearing three 2,6‐diaminopyridine and four terminal benzonitrile ligands (Figure 2).10e In addition, Bai, Guo, and Liu investigated the role of the metal by reacting benzonitrile with both a lithium and a sodium diamide, with Li giving increased yields of crystalline material (90 %) compared with the Na derivative (66 %).10d

**Scheme 3:**
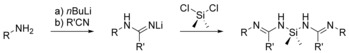
Synthesis of N‐bridged bis(amidine)s through route *v*). R=C(CH_3_)_3_, R′=C_6_H_5_.

Although silyl‐bridged bis(amidine)s are accessible through route *iv*), they are alternatively obtained by the reaction of lithiated amidines with chlorosilanes (Scheme 3).^13^ The one‐pot reaction starts with the lithiation of a primary amine, whose substituent will become the terminal group in the final bis(amidine). Subsequent reaction of the intermediary lithium amide with benzonitrile affords an lithium amidine complex, which, upon further treatment with Si(CH_3_)_2_Cl_2_, is converted to the bis(amidine). Although limited to one example, the yield is nearly quantitative.

**Scheme 4:**
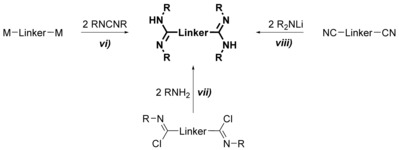
Synthesis of backbone‐bridged bis(amidine)s.

Backbone bridged bis(amidine)s (Scheme 4) are synthesized either: *vi*) by the addition of a carbodiimide to a doubly metallated compound,^14^ often generated in situ from the corresponding (di)halogen precursor, *vii*) through aminolysis of bis(imidoylchloride)s,14e, 15 or *viii*) by reacting a metallated amine with dinitriles.^16^

Carbodiimides readily insert into the metal–carbon bond of aromatic compounds or conjugated olefins and hydrolysis of the thus formed lithium bis(amidinate) complexes affords the free ligands in 30 to 72 % yield.^14^ So far, mostly diisopropyl‐ or dicyclohexylcarbodiimide have been applied to this reaction, likely due to the commercial availability of these two compounds, but reports on other carbodiimides, such as bis(2,6‐diisopropylphenyl)carbodiimide, start to arise. Although alkyl‐bridged bis(amidine)s have also been synthesized using this route, they are more readily available through the aminolysis of bis(imidoylchloride)s.14e, 15 This approach is the longest known and affords product yields ranging from 19 to 92 %, however, no general approach can be identified. Although the group of Beckert used a bis(imidoylchloride)amine stoichiometry of 1:2 along with triethylamine as an HCl scavenger,15b, 15f other protocols apply excessive amounts of the amine, thus acting as both, the nucleophile and the base. In either cases, comparable yields have been obtained thus the decision in favor of one over the other protocol is most likely driven by the boiling point of the amine used; for high‐boiling amines, such as bulky anilines, the method of Beckert and co‐workers appears to be preferential, whereas the opposite holds true for low‐boiling (aliphatic) amines. The generation of bis(imidoylchloride)s is circumvented when dicarboxylic acids are directly converted by the reaction with polyphosphoric acid trimethylsilyl ester (PPSE) and excess aniline as reported for 1,4‐cyclohexylene as well as 1,3‐ and 1,4‐phenylene bridged bis(amidine)s.15i, 17

**Scheme 5:**
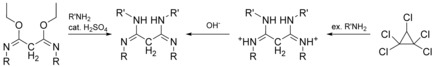
Synthesis of CH_2_‐bridged bis(amidine)s. R=CH(CH_3_)_2_, C(CH_3_)_3_.

CH_2_‐Bridged bis(amidine)s are also accessible on two alternative and rather unique pathways (Scheme 5). On the one hand, *N*,*N*′‐bis(4‐ethoxyphenyl)malonamides can be directly converted to the respective bis(amidine)s by treatment with various anilines in the presence of catalytic amounts of H_2_SO_4_,^18^ thus avoiding the transformation of the amide to an imidoylchloride. On the other hand, the reaction of pentachloro cyclopropane with an excess of alkylamine such as isopropylamine or *tert*‐butylamine, gives rise to bis(amidine)s in about 60 % yield.^19^

Finally, amidines, which carry an *m*‐terphenyl backbone that possesses benzaldehyde substituents have been bridged by condensation reactions of the aldehyde function using primary diamines,^20^ whereas amidines containing terminal alkyne groups have been linked through the *cis*‐ or *trans*‐coordination with platinum(II) centers^21^ or by Sonogashira and Glaser‐type coupling.^22^

The first report about a potential application of bis(amidine)s dates back to the 1950s,8a when researchers aimed for new local anesthetics based on phenacaine—an amidine—and intended that doubling the number of amidine units and separating them by a nontoxic linker gives rise to higher activities at a lower dosage. Although bis(amidine)s themselves have been pharmacologically tested,^18^ they found more often applications as supports for transition metals, rare‐earth and main‐group elements.^23^ The properties of the related complexes are strongly influenced by the way in which the two amidine units are bridged. In most of the cases, backbone‐bridged bis(amidine)s yield well‐defined complexes in which either one or two of the isolated amidine units are functionalized as discussed further below. The conformational flexibility of N‐bridged bis(amidine)s, however, offers access to mono‐ and polynuclear complexes, depending on the element, the electronic and steric properties of the ligand, and the synthetic protocol.

**Figure 3:**
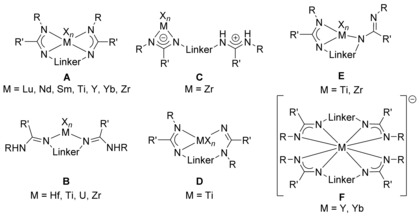
Types of mononuclear N‐bridged bis(amidinate) complexes.

**Figure 4:**
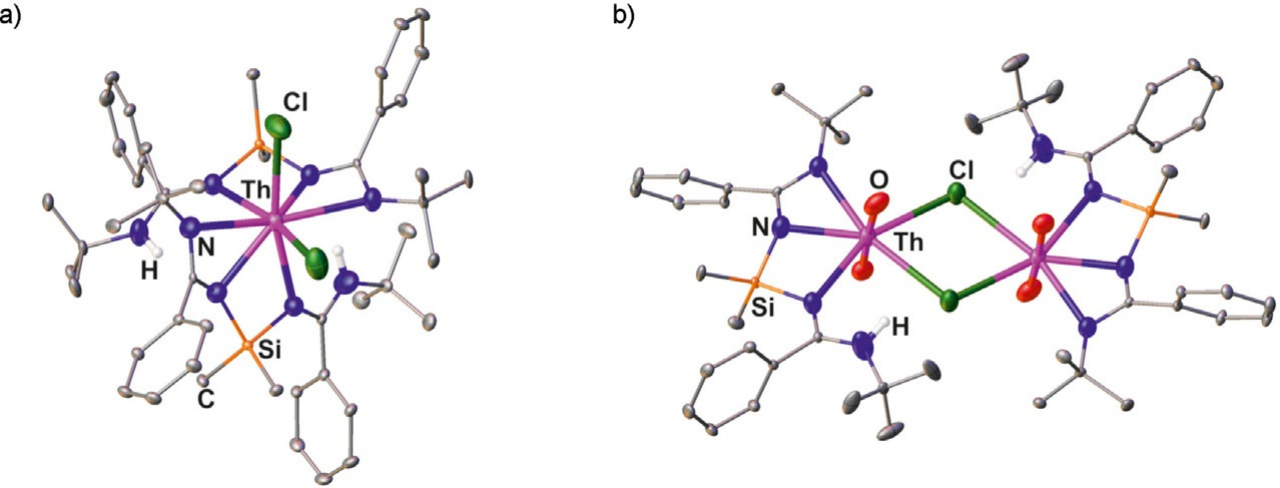
Molecular structures of a) **V** and b) **VI** in the solid state.27b Hydrogen atoms except those of the NH group and solvent molecules are omitted for clarity.

Monometallic complexes of type **A** are often observed when large ions such as Lu, Nd, Sm, Y, Yb, and Zr are involved (Figure 3),8f, 8g, 10a, 10e, 24 and the related bis(amidinate) complexes have found catalytic applications in hydrophosphonylation and ring‐opening polymerization reactions,24c, 24e, 24g but were also used as catalysts for the synthesis of monosubstituted N‐arylamidines.^25^ For smaller ions, however, only one example of a type **A** complex containing a titanium(IV) center is known to date.^26^ Mononuclear complexes of type **B**, in which the metal coordinates only the two lateral nitrogen atoms of each binding site, were observed when dimethylsilyl‐bridged bis(amidine)s are used for the complexation of hafnium, titanium, uranium, and zirconium, thus forming SiN_2_M four‐membered rings with M=Hf, Ti, U, Zr.^13^, 24h, 27 The coordination mode, however, depends not only on the metal but also on the solvent. For example, treating a dimethylsilyl‐bridged bis(amidine) (R=*t*Bu and R′=Ph) with ZrCl_4_ gives rise to either a complex (M=Zr, X=Cl, *n*=4) of type **B** or of type  **C** in 80 and 71 % yield, respectively, depending on whether toluene or dichloromethane is used as solvent.^13^ Furthermore, also the reaction conditions play a crucial role concerning the obtained products as exemplified with the reaction of a monolithiated bis(amidine) with thorium(IV) chloride: when dry THF has been used, the mononuclear species **V** was obtained whereas using wet THF gives rise to the dinuclear species **VI** (Figure 4).27b

**Figure 5:**
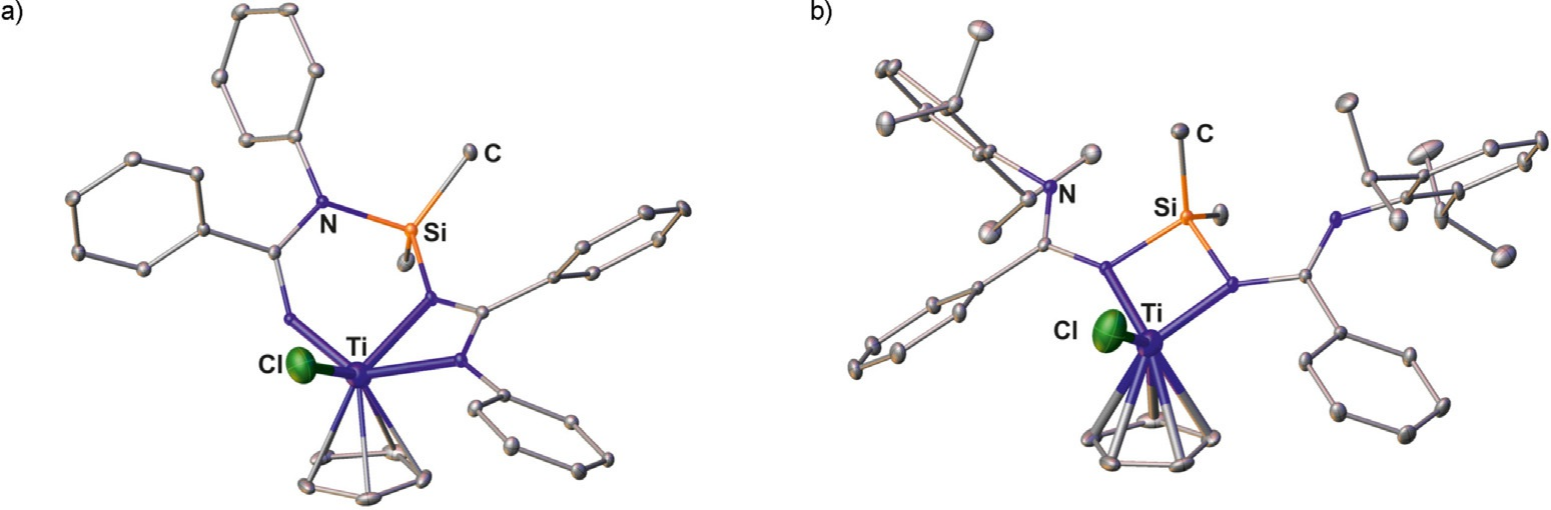
Molecular structures of a) **VII** and b) **VIII** in the solid state.27a Hydrogen atoms and solvent molecules are omitted for clarity.

Worthy of note is also the report about the transformation of a lithium bis(amidinate) to a heteroditopic imido amidinate complex (type **D**) upon reaction with TiCl_3_(C_5_H_5_), which however depends on the terminal rest. With less hindered phenyl or 2,6‐dimethylphenyl groups, the imido amidinate complex **VII** of type **D** is formed, whereas with the more bulky 2,6‐diisopropylphenyl substituent, the type **B** complex **VIII** was obtained (Figure 5).27a The coordination of only three out of four nitrogen atom is also observed in case of the type **E** complexes, in which the titanium and zirconium centers are bound to two lateral and one terminal nitrogen atom.24h

**Figure 6:**
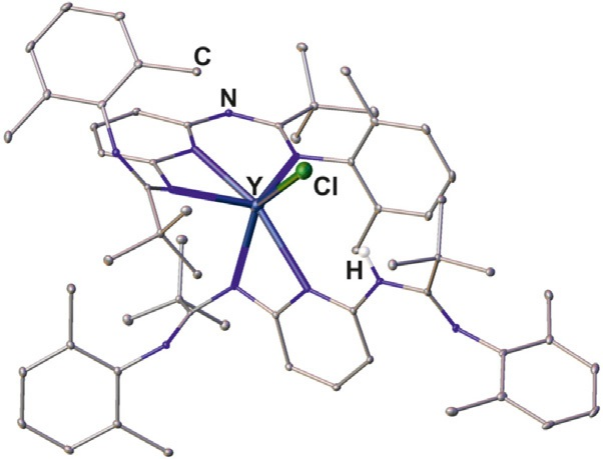
Molecular structure of **IX** in the solid state.^12^ Hydrogen atoms except those of the NH groups, counter ions and solvent molecules are omitted for clarity.

In addition, two mononuclear anionic complexes of type **F** have been reported, in which the lanthanide ion (Y, Yb) is eightfold coordinated to two bis(amidinate)s forming a trigonal dodecahedron.24c The catalytic activity of the mononuclear type **F** complexes in the ring‐opening polymerization of *ϵ*‐caprolacton was found to be lower than the activity of the respective dinuclear homoleptic complexes of type **I** (see further below). For the sake of completeness, it is worthy to note that with a 2,6‐pyridylene‐bridged bis(amidine) another type of mononuclear complexes has been reported for erbium and yttrium (**IX**, M=Y; Figure 6), in which two bis(amidine)s chelate one metal center involving coordination of the pyridyl nitrogen.^12^

**Figure 7:**
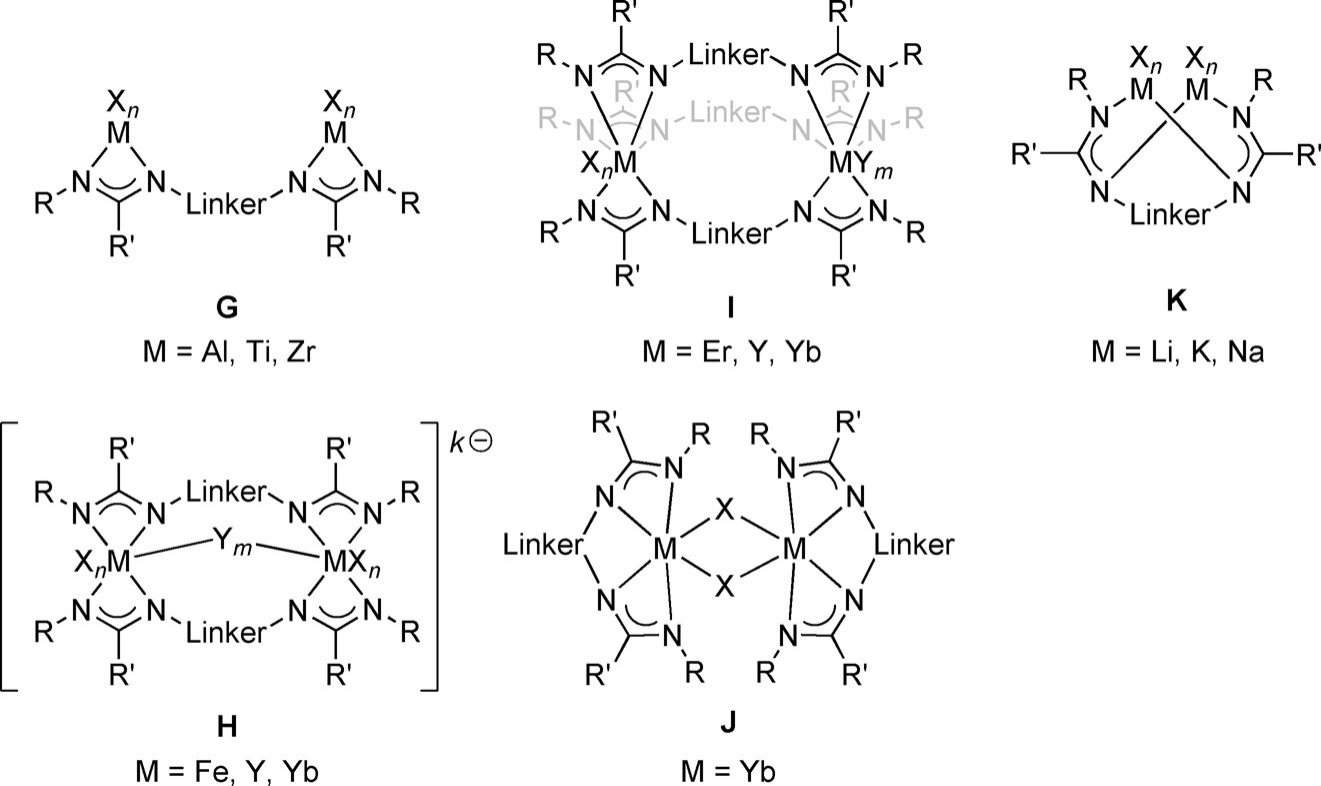
Types of dinuclear N‐bridged bis(amidinate) complexes.

In addition to the different types of mononuclear complexes, a variety of di‐ and also polynuclear bis(amidinate) complexes have been reported so far (Figure 7 and Figure 9, respectively). Heteroleptic dinuclear complexes of type **G** belong to the first observed bis(amidinate)s, but remain limited to certain examples employing the elements aluminum,7c, 9, 28 titanium,7a and zirconium.7b, 29 This is somewhat unfortunate keeping the promising catalytic ability of dinuclear aluminum (ring‐opening (co)polymerization of cyclic esters and synthesis of cyclic carbonates)^9^, ^28^ and zirconium complexes (stereoselective, living, coordinative chain‐transfer polymerization of propene)^29^ in mind. In addition, a variety of dinuclear complexes in which the metal centers are intra‐ or intermolecularly coordinated to two or three amidinate units have been reported, types **H**–**K**. For iron, neutral homoleptic type **H** complexes (*k*=*m*=*n*=0), in which the two paramagnetic iron(II) centers are framed by two bis(amidinate)s were observed,10b whereas for yttrium and ytterbium anionic and charge‐neutral complexes (*k*=0, 1) have been reported, for which additional bridging and/or terminal ligands/counterions are attached to the two metal centers.^12^, 24b, 24d, 30 These compounds are interesting catalysts, as shown for the charge‐neutral ytterbium type **H** complex, which initiates the polymerization of l‐lactide and *ϵ*‐caprolacton and possesses high activity along with good controllability for the ring‐opening polymerization.24b, 24d

**Figure 8:**
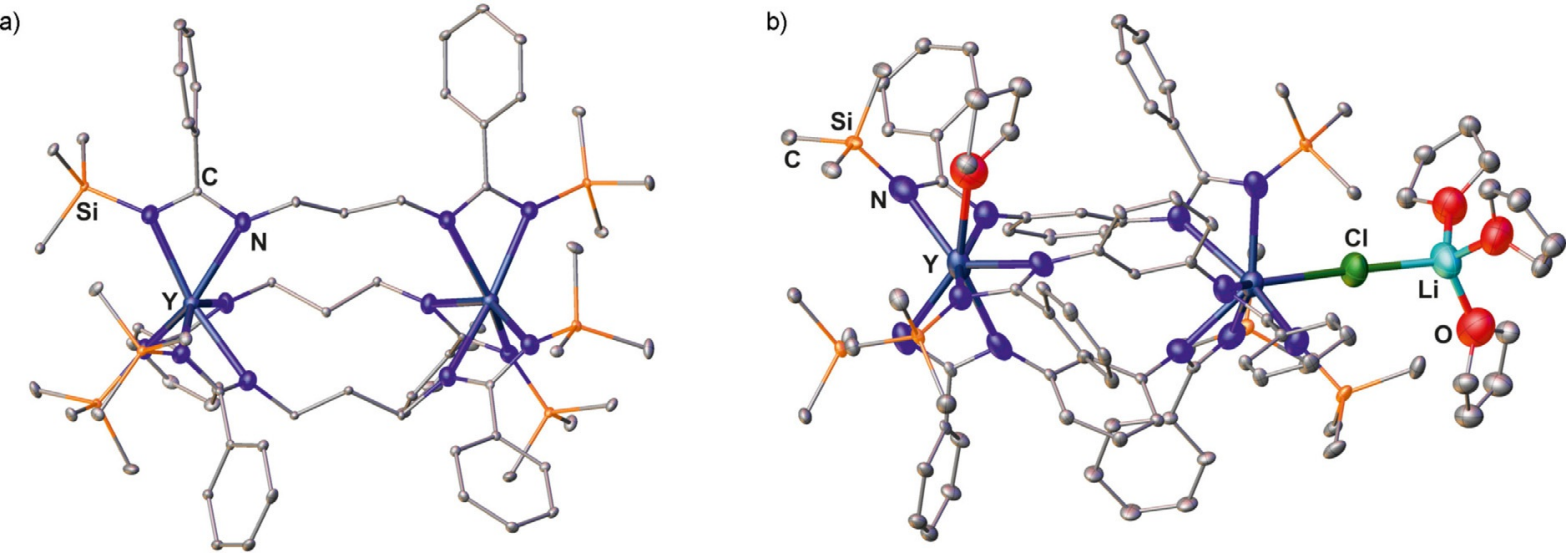
Molecular structures of a) **X** and b) **XI** in the solid state.^12^, 24c Hydrogen atoms and noncoordinated solvent molecules are omitted for clarity.

The coordination number of the two metal centers within complexes of type **I**, in which three bis(amidinate) ligands are heteroleptically bound to two metals (Er, Y, and Yb),^12^, 24c is strongly affected by the rigidity of the bridging group (Figure 8). For the flexible 1,3‐propylene linker, the purely homoleptic homodinuclear complex **X** with coordination number 6 (*m*=*n*=0) for each yttrium is obtained,24c whereas using a more rigid 1,3‐phenylene bridge yields the heterobimetallic complex **XI**, in which the two metals possess a coordination number of 7 (*m*=*n*=1) due to the additional complexation of one THF molecule and one Cl ion bridging the second yttrium and the lithium counterion, respectively.^12^ Although the purely homoleptic yttrium and ytterbium complexes bearing a 1,3‐propylene linker show higher activities in the ring‐opening polymerization of *ϵ*‐caprolacton than the respective mononuclear type **F** complexes, they yield polymers with rather broad molecular‐weight distributions, most likely because of several active lanthanide amidinate bonds. The aggregation of two mononuclear type **A** complexes gives rise to dinuclear type **J** complexes reported for ytterbium, and both complex types readily interconvert upon changing the solvent.24a Finally, the only other type of a dinuclear complex belongs to type **K**,8b, 8d, 10d in which the two metals coordinate crosswise one terminal and one lateral nitrogen atom of each amidinate unit as shown for examples of lithium, potassium, and sodium.

**Figure 9:**
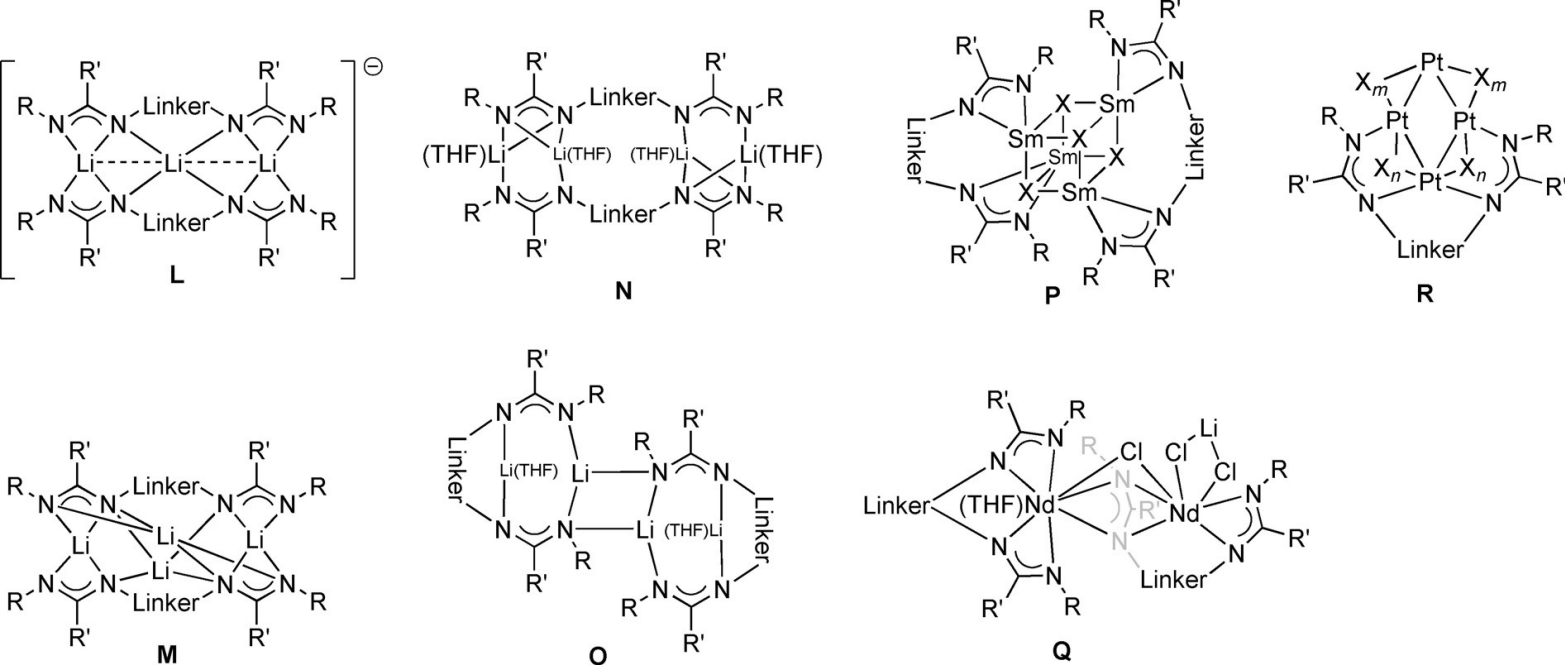
Types of polynuclear N‐bridged bis(amidinate) complexes.

**Figure 10:**
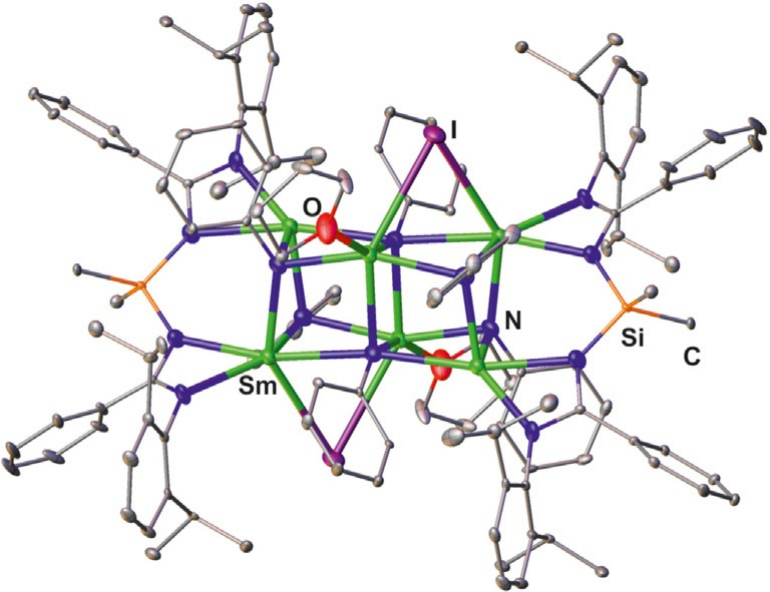
Molecular structures of **XII** in the solid state.32b Hydrogen atoms and noncoordinated solvent molecules are omitted for clarity.

In addition to the various dinuclear complex types **G**–**K**, also polynuclear complexes of the type **L**–**R** have been reported (Figure 9). The impact of the terminal substituent R on the coordination and aggregation behavior is nicely illustrated for dimethylsilyl‐bridged lithium bis(amidinate) complexes (R′=Ph). Although a dinuclear type **K** complex (X=THF, *n*=2) is obtained with terminal phenyl rests, increasing the steric bulk by using the 2,6‐diisopropylphenyl substituent affords a trinuclear type **L** complex, and terminal *tert*‐butyl groups give rise to a tetranuclear type **M** complex.10d Tetranuclear lithium complexes also show coordination modes of type **N** and **O**. In type **N** complexes,10e each lithium bridges the two bis(amidinate) units and reaches a coordination number of four due to the complexation of an additional THF molecule. The tetranuclear type **O** complexes, however, are formally generated upon aggregation of two dinuclear complexes, a behavior well‐known in the field of lithium chemistry as laddering principle;^31^ here, the lithium centers have a coordination number of three. For samarium, a tetranuclear cubic structure of type **P** 32 has been reported, in which the four Sm ions occupy alternating corners of the cube, whereas the other corners are occupied by μ_3_‐imido ligands (X=NPh). In addition, the doubly‐fused cubic samarium complex **XII**, consisting of six Sm centers, four μ_3_‐ and two μ_4_‐imido ligands as well as two bridging iodine ligands, has also been isolated (Figure 10).32b

Finally, two rather unusual complexes of type **Q** and **R** were reported: the former is a trinuclear heterobimetallic ate‐complex in which one bis(amidinate) chelates one neodymium through all four nitrogen atoms, whereas for the second bis(amidinate), both ligand sites act differently, that is one unit bridges both Nd centers whereas the second unit exclusively coordinates the second neodymium.10e A tetranuclear platinum complex of type **R** was formed in the reaction of a 1,3‐propylene‐bridged disodium bis(amidinate) with [Pt_4_(OAc)_8_]. Here, only three of the four platinum ions coordinate one bis(amidinate) ligand: one Pt bridges the two lateral nitrogen atoms and is bound to two Pt centers, which are each bound to one of the two terminal nitrogen atoms.8e

**Figure 11:**
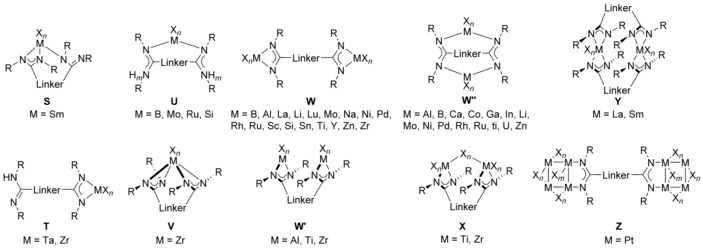
Types of mono‐ and dinuclear backbone‐bridged bis(amidinate) complexes.

**Figure 12:**
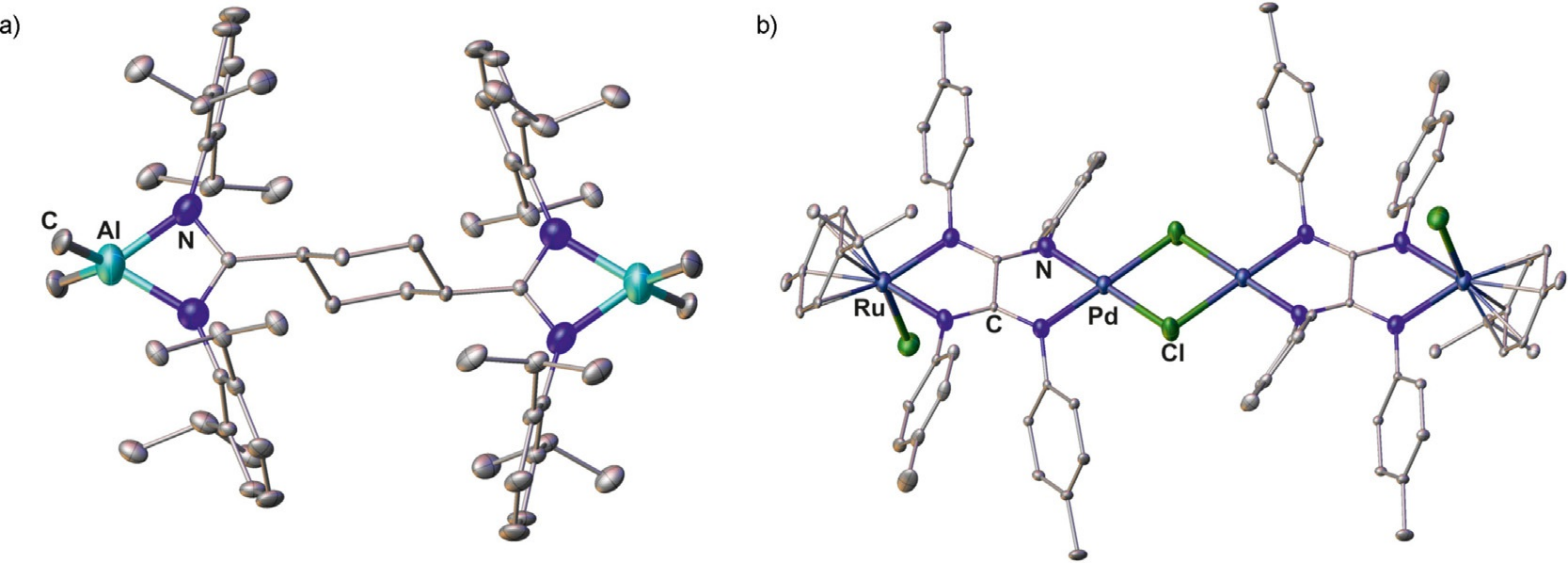
Molecular structures of a) **XIII** and b) **XIV** in the solid state.17b, 36 Hydrogen atoms and solvent molecules are omitted for clarity.

Compared with N‐bridged bis(amidinate)s, their backbone‐bridged relatives show a somewhat smaller set of known coordination modes (Figure 11). Due to the opposing orientation of both amidine units, most of the examples reported so far, belong to complexes of type **W**, which have well‐separated coordination sites and cover the elements B,^33^ Al,14c, 14d, 17b La,^34^ Li,14f Lu,14g, 14j, 17a, 34 Na,15i Rh,15i Sc,14g, 14j, 34 Ti,15c Y,14g, 14j, 17a, 34, 35 and Zr.15c The dinuclear aluminum complex **XIII** (Figure 12 a) serves as a representing example. Some of the type **W** complexes have been applied as catalysts for polymerization reactions: although complexes with rigid backbone‐bridges such as 1,4‐phenylene show no cooperative effects,^17^, ^34^ those with flexible bridges such as 1,4‐butylene excel their mononuclear counterparts in terms of activity and selectivity as illustrated in the living 3,4‐(co)polymerization of isoprene and myrcene.14j

**Figure 13:**
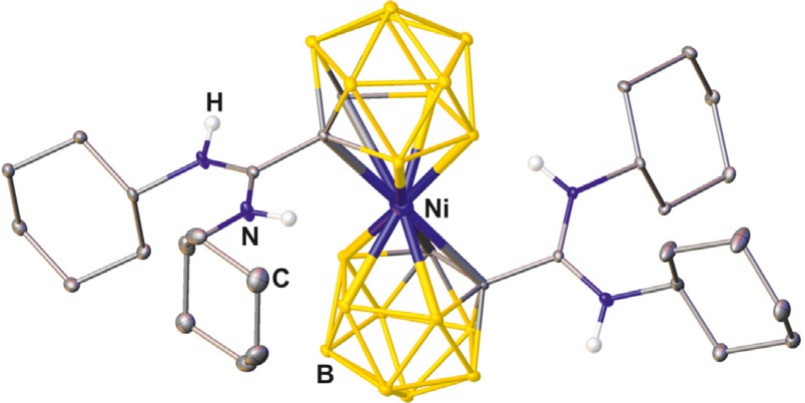
Molecular structures of **XV** in the solid state.^55^ Hydrogen atoms except those of the NH groups are omitted for clarity.

If the two amidine units are parallel or almost parallel oriented, which is given for backbone bridges like benzofuran, phenanthrene, or xanthene, the sub‐type **W**′ is derived and the examples known include aluminum,14e titanium,^37^ and zirconium.^38^ The parallel orientation eventually provides additional bridging groups to be installed as given in type **X** complexes (M=Ti,^37^, ^39^ Zr38b), but also allows for the isolation of a mononuclear complexes of type **V** in case of zirconium.38a Mononuclear complexes of type **S** have so far only been reported for one example containing samarium, which has been selectively synthesized by reacting a phenanthrene‐bridged bis(amidine) (R=isopropyl) with [Sm{N(SiMe_3_)_2_}_3_] in a 1:1 stoichiometry.14i Monometallic type **T** complexes incorporating Ta or Zr are also selectively synthesized in the same way,15c whereas with lanthanum and samarium dinuclear type **Y**‐complexes or even higher aggregates such as a tetranuclear lanthanum complex, are obtained.14i In addition, mononuclear complexes of type **U** are also known and examples incorporating B,^40^ Mo,^41^ Ru,^42^ and Si^43^ have been isolated so far. Finally, quite a few dinuclear type **W**′′ complexes have been reported in the literature, which are all based on oxalamidinates, and cover the elements Al,^44^ B,^45^ Ca,^46^ Co,^47^ Ga,^44^ In,^44^ Li,^48^ Mo,41a Ni,15d, 49 Pd,49b, 50 Rh,15i Ru,^42^ Ti,^51^ U,^48^ and Zn.^47^, 49b, 52 These are either directly obtained from the bis(amidine)15d, 42, 44, 45, 49b, 49c, 50b, 52 or from their alkali‐metal salts by salt metathesis.41a, 48, 50a In addition, the reductive coupling of carbodiimides utilized by organometallics,^51^ metallic lithium^48^ or LiUCl_4_ 48 also yields oxalamidinate complexes. In addition, coordination polymers of oxalamidinates have been reported49a, 49b, 50b and polymeric structures have been proposed for titanium(IV) species bridged by 1,4‐phenylene bis(amidinate)s.^53^ It is worth noting that also mixed type **W**′′ complexes of the couples Mo/Sn,41a Mo/Si,^41^ Mo/Ti41a Ni/Zn,49a, 49b Pd/Ru,^36^, ^54^ Pd/Zn49b, 50b are known and some of them form dimers through halogen bridges; the heterotetranuclear palladium ruthenium complex **XIV** (Figure 12 b) illustrates the molecular solid‐state structure. Finally, two rather unusual complexes are mentioned here for the sake of completeness: an octanuclear platinum complex of type **Z** 15h and the bis(3‐1,2‐carbollyamidinate)‐nickel(II) complex **XV** 55 (Figure 13), which is strictly speaking a bis(amidinium) complex although the nickel is chelated between two carbollide moieties.

## 3 Bis(guanidine)s

**Figure 14:**
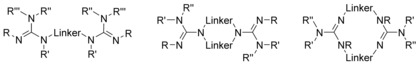
Acyclic, cyclic, and macrocyclic bis(guanidine)s.

Closely related to amidines are the more basic guanidines,^56^ whose donor capabilities are also increased compared with amidines due to the inclusion of an additional nitrogen atom in the backbone of the ligand.^57^ Bis(guanidine)s received considerable interest over the past decades,^58^ and have been used as catalysts,^59^ ion sensors,^60^ and superbases.^61^ In addition, they are used as ligands in bioinorganic and coordination chemistry.^62^ Bis(guanidine)s may be classified as acyclic, cyclic or macrocyclic compounds, depending on whether one or two linker groups are present in the molecule, Figure 14. Please note that, depending on the substituents R, R′, and R′′, several isomers (tautomers) are conceivable. Mono‐ (R=R′=R′′=H) and persubstituted (R or R′ or R′′≠H) bis(guanidine)s are beyond the scope of this review, but have been discussed elsewhere,58a, 58c, 63 wherefore compounds with one to three hydrogen atoms per guanidine unit are discussed in the following.

**Scheme 6:**
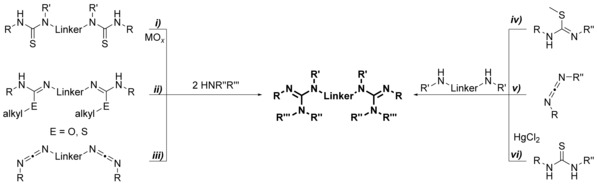
Synthesis of acyclic bis(guanidine)s.

**Scheme 7:**
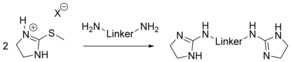
Synthesis of acyclic bis(aminoimidazoline)s.

Acyclic bis(guanidine)s are accessible on various routes as outlined in Scheme 6: *i*) from bis(thiourea)s using desulfurization agents,^64^ *ii*) from bis(*O*‐alkyl‐isourea)s or bis(*S*‐alkylisothiourea)s and amines,^65^ from *iii*) bis(carbodiimide)s^66^ or from the reaction of diamines with *iv*) N,N′‐disubstituted pseudothioureas,^67^ *v*) carbodiimides^68^ or *vi*) N,N′‐disubstituted thioureas and HgCl_2_.68c, 69 These routes give rise to bis(guanidine)s with a different degree of substitution: although using the routes *i*) to *iii*), di‐, tri‐, and tetra‐substituted bis(guanidine)s are accessible, on route *v*), di‐ and trisubstituted bis(guanidine)s have been prepared, and for the routes *iv*) and *vi*), all reports known to date are limited to trisubstituted compounds (R′=R′′=H). Overall, route *i*), a one‐pot reaction with a simple workup procedure, is preferential because the related bis(thiourea)s are readily available and the desulfurization agent PbO can be recycled.64b However, in case of ethylene‐bridged bis(thiourea)s bearing terminal alkyl groups, *N*‐(alkyl)‐4,5‐dihydro‐1*H*‐imidazol‐2‐amines are obtained instead of the desired bis(guanidine)s.64b If the terminal alkyl group is substituted by a sterically demanding aryl group, such as 2,6‐diisopropylphenyl, bis(guanidine)s are formed as a minor product, whereas 3,4‐ethylene‐bridged biguanides correspond to the main product.11b Noteworthily, bis(guanidine)s are also not accessible using the methods *ii*) and *iii*) because here intramolecular processes surpass the intermolecular reactions, giving rise to N,N′‐substituted *S*‐alkyl‐(4,5‐dihydro‐1*H*‐imidazol‐1)isothioureas and 3,4‐ethylene‐bridged biguanides, respectively.11b Alternatively, trisubstituted ethylene‐bridged bis(guanidine)s (R′=R′′=H) are readily available using route *iii*).68b Aminoimidazolines can be considered as the cyclic analogues of the guanidine group and the related bis(aminoimidazoline)s are readily available by treating primary diamines with 2‐alkylmercapto‐4,5‐dihydroimidazole salts (Scheme 7).68c, 70

Given that bis(phosphaguanidine)s are closely related to bis(guanidine)s, they are included here for the sake of completeness.^71^ Although there is only one report so far, the reaction of carbodiimides with dilithiated secondary diphosphines seems to be a suitable approach with yields of 61 to 69 %. In addition, phosphaguanidines give rise to the related bis(phosphaguanidine)s upon coordination with copper(I) or platinum(II).71b Cyclic bis(guanidine)s are reminiscent of backbone‐bridged bis(amidine)s (see above) and readily available through the reaction of cyclic diamines with carbodiimides, (route *v*), R′=Linker).58b, 72 Alternatively, they can be obtained through the aminolysis of chloroamidines^73^ or by treating cyclic diamines with *N*,*N′*‐di‐Boc‐*N*′′‐triflylguanidine.^74^

**Scheme 8:**
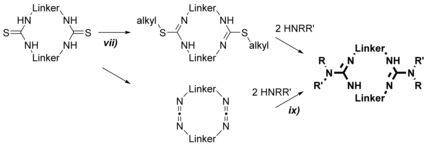
Synthesis of macrocyclic bis(guanidine)s.

To derive macrocyclic guanidines, two methods are conceivable for their synthesis (Scheme 8). Regarding the first route (*vii*), a macrocyclic bis(thiourea) is transferred to the respective bis(pseudothiourea) by S‐alkylation and subsequently treated with two equivalents of a primary or secondary amine.^59^, 60a In the second approach (*ix*), a bis(carbodiimide), which is either generated from the bis(thiourea) or from bis(iminophosphorane), is reacted with an excess of amine yielding the related macrocyclic bis(guanidine)s in very good yields.^75^

**Figure 15:**
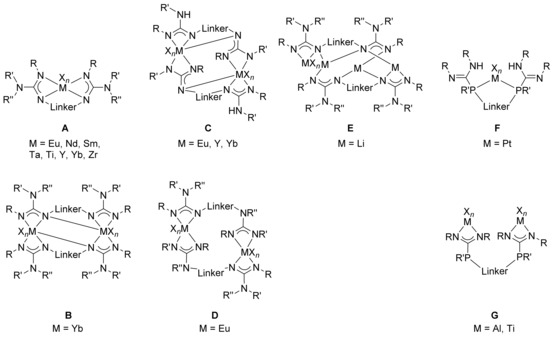
Types of mono‐ and polynuclear bis(guanidate) **A**–**E** and bis(phosphaguanidate) **F**–**G** complexes..

**Figure 16:**
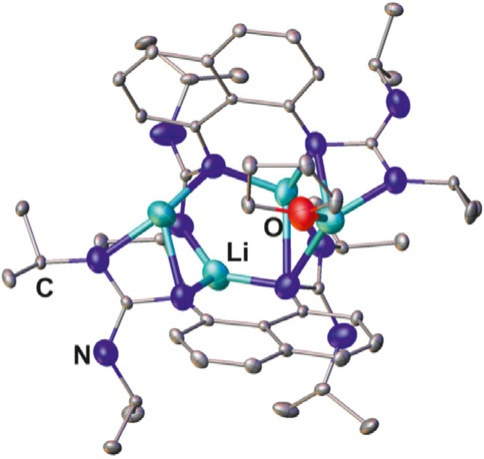
Molecular structures of **XVI** in the solid state.^81^ Hydrogen atoms are omitted for clarity.

Compared with bis(amidine)s, bis(guanidine)s found multifarious applications as catalysts,^59^, ^76^ ion sensors,^60^ pharmaceuticals,^63^ or superbases.^61^ To some extent, bis(guanidine) complexes (Figure 15) resemble the coordination patterns observed for bis(amidinate)s. Mononuclear complexes of type **A** are known for Eu,^77^ Nd,^78^ Sm,^79^ Ta,^80^ Ti,68b Y,^78^, ^81^ Yb,^78^, ^81^, ^82^ Zr,68b and have been either directly derived from the bis(guanidine)68b or from in situ generated dinuclear alkali‐metal complexes,^77^, ^78^, ^79^, ^80^, ^81^ which are readily accessible from the reaction of metallated secondary diamines with two equivalents of a carbodiimide. Due to the additional nitrogen‐donor function compared with bis(amidine)s, further coordination modes have been reported for dinuclear complexes of rare‐earth metals. Although type **B** complexes were only obtained for Yb,^81^ complexes of type **C** have been characterized for Eu,^77^ Y, and Yb,^81^ and only one complex of type **D** is known, in which M=Eu.^77^ Finally, for lithium, also the tetranuclear type **E** complex **XVI** has been reported (Figure 16) in which three different coordination modes have been observed for the lithium ions, although they all have the coordination number three.^81^ Notably, bis(phosphaguanidine)s show different coordination schemes depending on the metal involved. Platinum(II) coordinates to the two lateral phosphorus atoms yielding mononuclear complexes of type **F**,71a whereas with aluminum and titanium, dinuclear type **G** complexes are obtained in which the two metals are coordinated in an N,N‐chelating fashion.71b, 71c

## 4 Bis(β‐diimine)s

**Figure 17:**
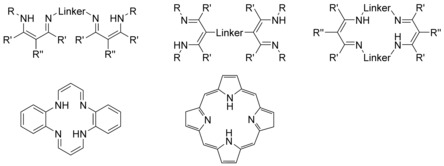
Acyclic and macrocyclic bis(β‐diimine)s.

**Scheme 9:**
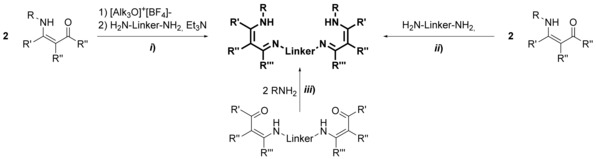
Synthesis of acyclic N‐bridged bis(β‐diimine)s.

Given that β‐diiminate (NacNac) ligands have been identified as powerful supports in the 1960,^83^ they are commonly used for the stabilization of elements in various oxidation states and from all sections of the periodic table.^84^ In the following, the term β‐diimine will be used although the terms β‐diketimine or 1,3‐diimine are also used in the literature. Please note that, the substituents have a strong influence on whether the amino–imine (shown in Figure 17) or the β‐diimine tautomer represent the most stable isomer. Due to stabilization by hydrogen bonding between the imine nitrogen atom and the N−H proton, the ligands discussed in this chapter exist by and large as the amino–imine tautomer. However, and because of the frequent use of the term bis(β‐diimine) in the literature this term will be used in here as well. Among the bis(β‐diimine)s, different kinds occur in the literature, these being the N‐bridged and backbone‐bridged acyclic as well as macrocyclic derivatives (Figure 17). The latter motif is not only regularly found in natural macrocycles such as bacterchlorin, chlorin, isobacterchlorin, porphodimethane, and porphyrin,^85^ but also in tetraazaanulenes,^86^ which have been discussed elsewhere and will remain undiscussed in here. Acyclic backbone‐bridged bis(β‐diimine)s were first reported in 2004 by the Lappert group^87^ and the first N‐bridged example with isolated binding sites was presented one year later by the group of Hultzsch.^88^ Since then, three methods have been successfully applied for the synthesis of N‐bridged bis(β‐diimine)s (Scheme 9). Although two of them start from β‐aminoketones, a successful, neat reaction is only observed with 1,3‐bis(aminomethyl)benzene (route *ii*).^89^ In case of other diamines, pre‐activation of the β‐aminoketone using trialkyl oxonium tetrafluoroborate, also known as Meerwein's salt,^90^ is necessary.^88^, ^91^ All derivatives reported so far contain terminal aryl groups, whereas the opposite holds true for the first compartmental N‐bridged bis(β‐diimine), containing an additional amine group in the bridge, which was obtained through route *iii*) by treating a bis(β‐aminocarbonyl) compound with primary alkyl amines.^92^

**Scheme 10:**
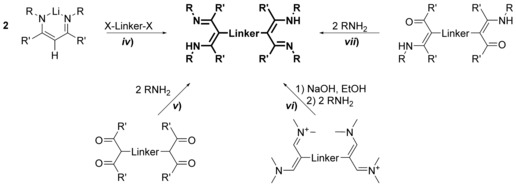
Synthesis of acyclic backbone‐bridged bis(β‐diimine)s.

Bis(β‐diimine)s with various backbone‐substituents, including alkyl,^87^ aromatic,^93^ and disulfide^94^ bridges, are known today. The synthetic procedures differ to quite some extent (Scheme 10) depending on the nature of the linker group. An alkyl‐bridged bis(β‐diimine) is obtained when two equivalents of a lithium β‐diiminate are allowed to react with one equivalent of an alkyl dihalide (route *iv*).^87^ For aromatic bridges, two different routes have been applied: route *v*) starts from bis(β‐dione)s and anilines,93c whereas route *vi*) follows a rather complex sequence in which a bis(vinamidinium) salt is hydrolyzed and subsequent reaction with an aniline yields the desired bis(β‐diimine).93a, 93b Finally, disulfide‐bridged derivatives are obtained, when a bis(β‐aminocarbonyl) species is allowed to react with anilines (route *vii*).^94^

**Scheme 11:**
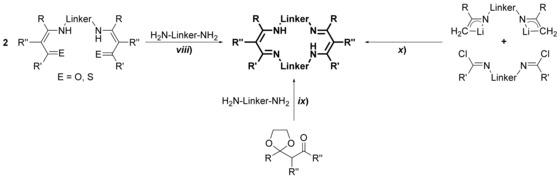
Synthesis of macrocyclic bis(β‐diimine)s.

Macrocyclic bis(β‐diimine)s often belong to tetraazaanulenes, which are regularly obtained through the related transition‐metal bis(β‐diiminate) complexes by applying template synthesis,^95^ but are outside this review. Apart from them, other types of macrocyclic bis(β‐diimine)s have been reported, which originate from the condensation of primary diamines with *viii*) bis(β‐aminocarbonyl)^96^ and bis(β‐aminothiocarbonyl) compounds,^97^ respectively, or *ix*) the ethylene glycol monoketal of β‐diones.^98^ Alternatively, macrocyclic compounds are also formed in the reaction of metallated bis(imine)s and bis(imidoylchloride)s (route *x*, Scheme 11).96c

**Figure 18:**
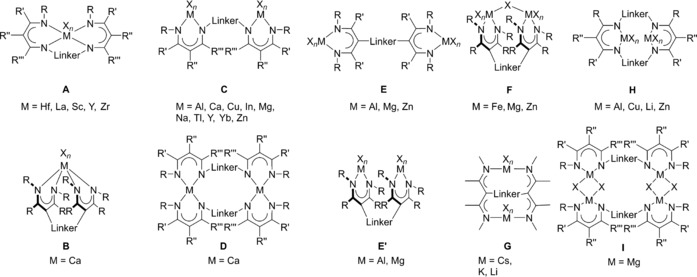
Types of bis(β‐diiminate) complexes.

So far, bis(β‐diimine)s have not found applications by themselves, but have been used as supports for transition metals as well as rare‐earth and main‐group elements, yielding mono‐ and dinuclear metal complexes (Figure 18). For mononuclear complexes of type **A**, incorporating Hf,91d La,^88^, 91a, 91c Sc91g Y,^88^, 91a, 91c, 91g and Zr,91d, 91f several isomers exist, which show a fluxional behavior and interconvert through a Bailar twist processes. It could be shown that some of the rare‐earth complexes were moderately active in the copolymerization of cyclohexene oxide (CHO) with carbon dioxide.91a With (almost) parallel oriented NacNac moieties, a mononuclear complex of type **B** has been isolated, in which a calcium is framed by both bis(β‐diiminate) units and a coordination number of five is reached through the complexation of an additional THF molecule.^99^

**Figure 19:**
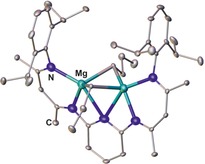
Molecular structures of **XVII** in the solid state.103b Hydrogen atoms are omitted for clarity.

Heteroleptic dinuclear complexes of type **C** are known for transition metals (Cu,91h Y,^100^ Zn91b, 91e, 91j) as well as for rare‐earth (Yb)^100^ and main‐group elements (Al,^101^ Ca,91b, 91e In,^102^ Mg,^103^ Na,^100^ Tl^89^, ^102^). Notably, calcium and magnesium complexes of type **C** show a dynamic behavior and undergo rapid ligand exchange in accordance with the Schlenk equilibrium yielding homoleptic complexes of type **D**.91b, 91e, 103c The equilibrium, however, is strongly affected by the bridge. The linker group also plays a crucial role if it contains additional donor functions. Thus, using a 2,6‐pyridylene bridge leads to dimerization,91h yields a hexanuclear copper complex,^104^ or incorporates the pyridyl nitrogen atom into the coordination sphere of the two magnesium or zinc centers91b, 103b as illustrated for the dinuclear magnesium complex **XVII** (Figure 19).

**Figure 20:**
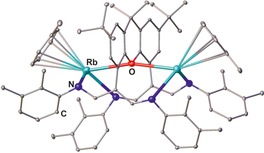
Molecular structures of **XVIII** in the solid state.^105^ Hydrogen atoms and noncoordinating solvent molecules are omitted for clarity.

The catalytic activity of some dinuclear type **C** complexes in the copolymerization of CHO and CO_2_ has been evaluated by the group of Harder. Although calcium complexes remained inactive, several zinc complexes efficiently catalyzed the polymerization and the activity found to be highly dependent on the nature of the bridging unit, with 1,3‐phenylene being a better choice compared with 1,4‐phenylene, whereas complexes containing a hydrazine bridge were found to be inactive.91b, 91e Three variants of backbone‐bridged bis(β‐diiminate)s have been so far reported in the literature. The heteroleptic complexes of type **E** are known for aluminum^87^, ^105^ magnesium,^105^ and zinc,93b whereas examples of type **E**′ complexes are known for aluminium and magnesium.^99^, ^105^ Type **F** complexes are obtained when an additional ligand such as chlorido or *n‐*butyl bridges the two metals (M=Fe, Mg, Zn) of complex **E**′.91b, 103b, 106 Finally, also homoleptic backbone‐bridged cesium, lithium, and potassium complexes of type **G** are known, which nicely illustrates the effect of ligand and metal as the related aluminum, calcium, and magnesium complexes exists as type **B**, **E**, and **E**′ complexes, respectively.^87^, ^99^ Regarding the N‐bridged derivatives, an additional donor site within in the linker group affects the coordination behavior. Thus, the unusual dinuclear complex **XVIII** (Figure 20), in which each rubidium center is coordinated to only one nitrogen atom per (β‐diiminate) unit and the oxygen atom of the xanthene bridge, has also been reported.^105^ Macrocyclic bis(β‐diiminate) type **H** complexes have been reported for Al,^98^ Cu,96a Li,^98^ and Zn,^96^, ^98^ and the rigidity of the ligand framework defines not only the metal–metal separation but also the relative orientation of both metal centers. The zinc derivatives are of particular importance because these are valuable catalyst for the copolymerization of CHO and CO_2_.^96^, ^98^ Finally, tetranuclear complexes of type **I** are formed upon dimerization of heteroleptic dinuclear type **C** magnesium complexes.103d, 103e

## 5 Bis(aminotroponimine)s

**Scheme 12:**
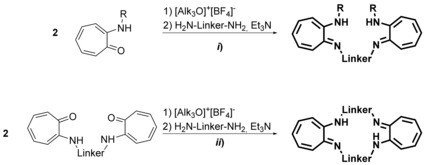
Synthesis of N‐bridged and macrocyclic bis(aminotroponimine)s.

Aminotroponimines belong to an interesting class of ligands, which exhibit a 10‐electron π‐system delocalized over seven carbon and two nitrogen atoms, and recently experienced a renaissance in coordination chemistry.^107^ Their macrocyclic (tropocoronands) or N‐bridged relatives have been introduced by the groups of Lippard^108^ and Roesky,^109^ respectively, but after a period of intense research,^110^ interest in this compound class faded almost completely. The synthesis of bis(aminotroponimine)s resembles to a certain extent the synthesis of bis(β‐diimine)s (see above). Activation of aminotropones (route *i*) or bis(aminotropone)s (route *ii*)^108^ by Meerwein's salt and subsequent treatment with a primary diamine gives rise to the related N‐bridged or macrocyclic bis(aminotroponimine)s, respectively (Scheme 12). Alternatively, Meerwein's salt maybe substituted by dimethylsulfate in route *ii*).110c

**Figure 21:**
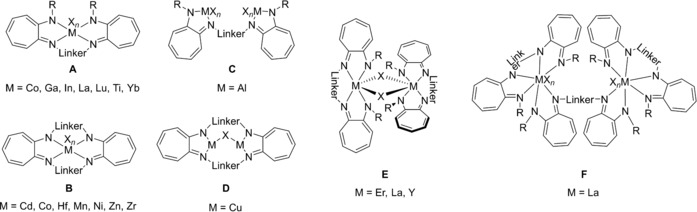
Types of bis(aminotroponiminate) complexes.

**Figure 22:**
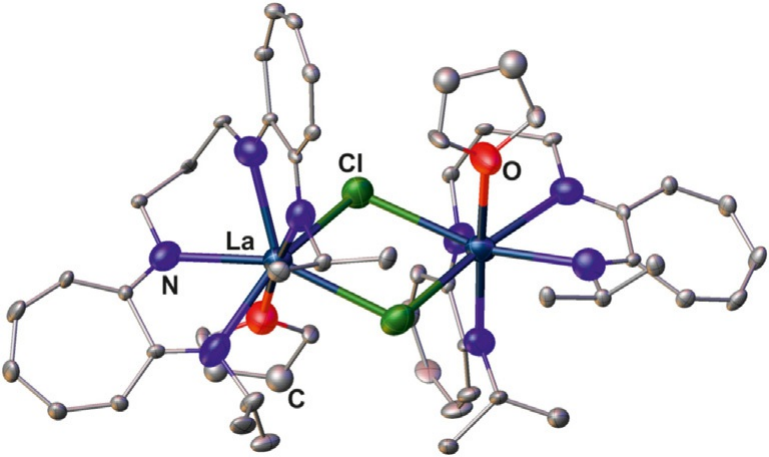
Molecular structures of **XIX** in the solid state.^109^ Hydrogen atoms and noncoordinating solvent molecules are omitted for clarity.

As in case of the bis(β‐diimine)s, bis(aminotroponimine)s have solely been applied as supports in various metal complexes (Figure 21). Most examples are known for mononuclear complexes of either type **A**, for M=Co,110m Ga,110l In,110l La,^109^ Lu,110n Ti,110i Yb,110n or type **B**, for Cd,110f Co,110e, 110p Hf,110d Mn,110g Ni,^108^, 110a Zn,110f Zr,110d depending on whether N‐bridged or macrocyclic bis(aminotroponimine)s being applied. In case of fourfold‐coordinated metal centers, the linker group strongly affects the conformation of the related complex, specifically being distorted in planar and tetrahedral manner. In case of hexacoordinated metal centers, the linker group also impacts the overall stereochemistry. Surprisingly, the coordination schemes are less manifold compared than for ligands reported in the preceding chapters and there are only very few examples of dinuclear complexes of type **C** (M=Al)110k and **D** (M=Cu),110q in addition to the rather unusual complexes of type **E** (M=Er,110h La,^109^ Y)110h and **F** (M=La);^109^ the dinuclear lanthanum complex **XIX** illustrated in Figure 22 stands as an example.

## 6 Bis(pyrrolimine)s

**Scheme 13:**
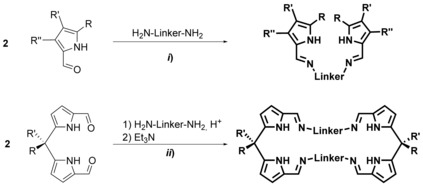
Synthesis of N‐bridged and macrocyclic bis(aminotroponimine)s.

Acyclic bis(pyrrolimine)s are readily accessible in a one‐pot reaction from commercial starting materials, this is a substituted 1‐*H*‐pyrrole‐2‐carboxaldehydes and primary diamines (route *i*, Scheme 13).^111^ Over the last two decades, a variety of macrocyclic derivatives have been introduced and widely applied in coordination chemistry. Although these macrocycles are formally tetra(pyrolimine)s, they are mentioned here for the sake of comparison but will not be discussed in detail.^112^ Their synthesis originates from diiminodipyrromethanes, which are readily obtained through cyclization with primary diamines under acidic conditions and workup with a suitable base, liberating the free ligands (route *ii*, tropocoronands).

**Figure 23:**
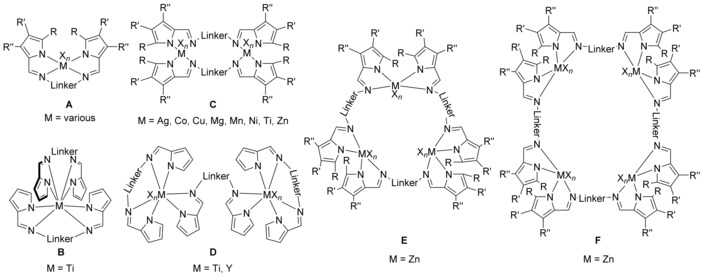
Types of bis(pyrroliminate) complexes.

**Figure 24:**
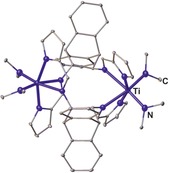
Molecular structures of **XX** in the solid state.^120^ Hydrogen atoms and solvent molecules are omitted for clarity.

The facile synthesis of acyclic bis(pyrrolimine)s most likely accounts for their manifold application giving rise to various mono‐ and polynuclear coordination compounds (Figure 23). Hundreds of examples have been reported for mononuclear complexes of type **A**, for example for Cu,^113^ Mn,^114^ Ni,113b Y,^115^ Zr,^116^ and listing them in here would exceed the limits of this review. In contrast, only one example is known for a type **B** complex, in which the titanium is eightfold coordinated by two bis(pyrroliminate)s forming a trigonal dodecahedron as reported for the bis(amidinate) complex of type **E** (see above).^117^ Dinuclear bis(pyrroliminate) compounds have also been reported, and homoleptic type **C** complexes could be isolated for Ag,111b Co,^118^ Cu112c, 113a, 119 Mg,^116^ Mn,^114^ Ni,112c Ti,^120^ and Zn.113b, 116, 121 Here, various configurations are conceivable and most often, the complexes are intertwined, forming helix‐type structures. Among them, the dinuclear titanium complex **XX** (Figure 24) did show catalytic activity for the polymerization of *rac*‐lactide^120^ and the cobalt and manganese complexes were found to readily activate O_2_.^114^, ^118^ When three ligand molecules frame two metals, dinuclear complexes of type **D** are obtained. Examples of titanium^117^ and yttrium^115^ species have been reported and the titanium complex catalyzes the hydroamination reaction of phenylacetylene with aniline under mild conditions. In addition, trinuclear (type **E**) and tetranuclear (type **F**) zinc complexes have also been observed and the examples nicely illustrate how the bridging group impacts the overall coordination mode.121a Although with a 1,2‐phenylene bridge a type **C** complex is obtained, a 1,3‐phenylene bridge allows for the isolation of the trinuclear complex **E** and using a 1,4‐phenylene bridge finally gives rise to a tetranuclear complex of type **F**.

## 7 Miscellaneous

**Figure 25:**
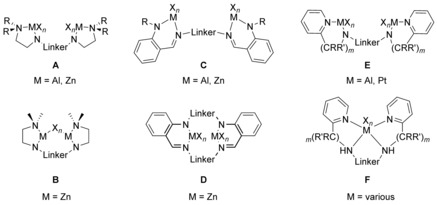
Complexes of miscellaneous nitrogen‐based ligands.

In addition to the various ditopic ligands mentioned in the preceding chapters, some additional N,N‐ligands are worthy to mention even though they found only little precedence in the literature so far; their metal complexes are shown in Figure 25.

**Figure 26:**
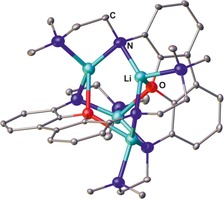
Molecular structures of **XXI** in the solid state.^123^ Hydrogen atoms and solvent molecules are omitted for clarity.

In comparison with amidines, guanidines, β‐diimines, and aminotroponimines, amidoamines offer some advantages.^122^ Their saturated backbone suppresses the delocalization and inhibits a non‐innocent behavior, which is of particular importance as in dinuclear complexes ligand‐centered reactions are regularly observed. Bis(amidoamine)s reported so far, were obtained by Cu‐catalyzed coupling of diiodide species with N,N‐alkylated ethylenediamines^123^ and readily transferred to the related aluminum and zinc complexes of type **A** and **B**.^123^, ^124^ The deprotonation of bis(amidoamine)s with *n‐*butyllithium affords the tetranuclear complex **XXI** (Figure 26) in which the oxygen atoms of the benzofuran bridge are also involved in the coordination scheme.^123^ Bis(anilidoaldimine)s are an additional class of ligands, which are accessible in excellent yields from the reaction of diamines/dianilines with fluorobenzaldehydes and subsequent nucleophilic aromatic substitution of the fluorine groups with two equivalents of a lithium anilide.^125^ The macrocyclic derivatives cannot be obtained on this route, but reacting the dianiline with 1,3‐dioxolane‐protected 2‐bromobenzadehyde followed by deprotection and cyclization with the HCl salts of the dianiline yields the desired macrocycle. The related type **C** (Al, Zn)^125^ and **D** (Zn)125a complexes are readily obtained upon treatment of the protio ligands with aluminum and zinc alkyls, respectively, and behave as active catalysts for the copolymerization of CO_2_ and epoxides. Bis(aminopyridine)s have been used recently and give rise to manifold coordination complexes. The protio ligands are readily accessible by reducing the related bis(iminopyridine)s with NaBH_4_.^126^ Although aluminum complexes, which were accessed by reacting the related bis(iminopyridine)s with trialkyl aluminum, were characterized as type **E** complexes by X‐ray diffraction,^127^ for platinum this coordination mode was proposed based on computational calculations.126b, 126c Notably, most of the complexes incorporating bis(aminopyridine)s belong to mononuclear coordination complexes of type **F** in which the ligand remains in its neutral protonated form, thus being beyond the scope of this review.^128^

## 8 Conclusions and Outlook

Over the last decades, whole libraries of nitrogen‐based ditopic ligands have been discovered and applied in various fields of chemistry. The multifarious coordination modes originating from these ligands are challenging, in terms of predicting and manipulating the coordination schemes by a proper choice of backbone, bridging group, terminal substituents, and the metal(loid) itself. Similarly, interesting coordination compounds reaching from mononuclear to polynuclear complexes have been reported and especially the latter are of particular interest for the rapidly growing field of cooperative catalysis, which requires suitable ligands that allow to finetune both the metal–metal separation and the relative orientation of both active sites. In addition to being valuable supports for one or more metal centers, the ligands themselves might be interesting catalysts or materials with properties not observed for their monotopic counterparts.

## Conflict of interest

The authors declare no conflict of interest.

## Biographical Information

*Robert Kretschmer studied chemistry in Jena and obtained his Ph.D. at the TU Berlin in 2012 under the guidance of Prof. Helmut Schwarz. After a postdoctoral stay with Prof. Guy Bertrand at the UCSD*, *he started his independent career in 2015 at the University of Regensburg and in 2019 he became Junior Professor with Tenure Track at the Friedrich Schiller University Jena. His research focusses on ligand design and cooperative effects originating from polynuclear systems and is funded by the German Science Foundation (DFG). In 2017 he became an elected Member of the German Young Academy (Die Junge Akademie*.

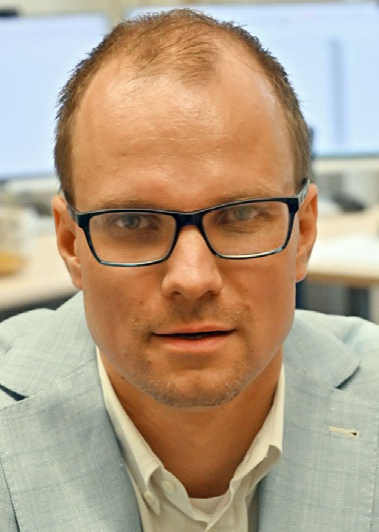
